# A Smart Mutation Scheme Produces Hundreds of Functional Proteins

**DOI:** 10.1371/journal.pbio.0040136

**Published:** 2006-04-11

**Authors:** Richard Robinson

A protein's structure dictates its function, and one of the most direct and powerful ways to explore a protein's function is by modifying its structure. Such an exploration is carried out naturally every time a protein's gene is mutated, and the same process can be mimicked in the lab. Unfortunately, approaches that introduce random mutations frequently disrupt the interactions that keep a protein properly folded, rendering the mutant entirely functionless and the experimental results largely uninformative about the contributions made by specific amino acids to overall function. In a new study, Christopher Otey, Frances Arnold, and colleagues use recombination guided by structural modeling to efficiently generate a family of thousands of properly folded mutants of a protein, and reveal previously unknown influences on the protein's function.

The authors studied versions of the protein cytochrome P450—a diverse protein family whose members govern a host of cell reactions, including detoxifying drugs and aiding construction of a wide variety of complex molecules. Each cytochrome P450 has nestled within it a heme group, a two-dimensional cage for an iron atom that is also found at the heart of hemoglobin. To generate the new cytochromes, they used a structural analysis tool called SCHEMA, which identifies how to divide multiple cytochromes into smaller “blocks.” Reassembling these blocks creates chimeras that have a good chance of being functional, but which, at the same time, have large numbers of mutations (up to 109) compared with the parent sequences. Otey et al. modeled the consequences of millions of such mutations, and chose a set of blocks that, according to the analysis, retained the greatest number of contacts between amino acids in the protein, reasoning that this would increase the likelihood of proper folding. Moving from computer to Petri dish, they then generated a set of over 6,000 new genes and expressed them in bacteria.

From the bacterial colonies, each with a unique cytochrome P450, they randomly chose almost 1,000 for detailed analysis. In about half of these, the protein folded properly and bound its heme group, despite differing from the naturally occurring proteins at 70 out of about 460 amino acid positions on average. Of those that folded correctly, about three-quarters were also functionally intact, able to catalyze reactions similar to those of the native proteins. Detailed analysis revealed the contribution to catalytic activity of a previously unappreciated amino acid near the enzyme's active site. Some of the mutants were more resistant to heat degradation than the originals, indicating the potential for creating novel and possibly useful new variants using this method.

The mutation method used here increased the yield of properly folded cytochromes by 10,000-fold over entirely random mutation techniques, and in one fell swoop nearly doubled the number of extant functional cytochromes P450. The results of this study will be useful for further structural analysis of the cytochrome P450 family, and the SCHEMA method for generating structurally intact mutants is likely to be applied to other protein families as well, both to tease out their structural secrets and perhaps to generate proteins with new properties that could be exploited for commercial or medical applications.

## 

**Figure:**
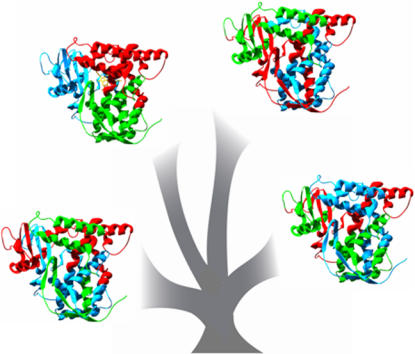
The creation of an artificial family of thousands of new proteins by shuffling protein building blocks allows us to probe protein structure and function, free from the filtering effects of natural selection.

